# A Case of Hypoglycemia Diagnosed by Neuropsychiatric Symptoms after Distal Gastrectomy and Total Colectomy

**DOI:** 10.70352/scrj.cr.24-0099

**Published:** 2025-03-12

**Authors:** Kota Okuno, Masahiro Niihara, Shohei Fujita, Tadashi Higuchi, Hiroki Harada, Marie Washio, Mikiko Sakuraya, Koshi Kumagai, Takeo Sato, Takafumi Sangai, Yusuke Kumamoto, Takeshi Naitoh, Keishi Yamashita, Naoki Hiki

**Affiliations:** 1 Department of Upper Gastrointestinal Surgery, Kitasato University School of Medicine, Sagamihara, Kanagawa, Japan; 2 Digestive Disease Center, International University of Health and Welfare, Mita Hospital, Tokyo, Japan; 3 Department of Lower Gastrointestinal Surgery, Kitasato University School of Medicine, Sagamihara, Kanagawa, Japan; 4 Department of Breast and Thyroid Surgery, Kitasato University School of Medicine, Sagamihara, Kanagawa, Japan; 5 Department of General-Pediatric-Hepatobiliary Pancreatic Surgery, Kitasato University School of Medicine, Sagamihara, Kanagawa, Japan; 6 Division of Advanced Surgical Oncology, Research and Development Center for New Medical Frontiers, Kitasato University School of Medicine, Sagamihara, Kanagawa, Japan

**Keywords:** hypoglycemia, CGM, continuous glucose monitoring, CIPO, chronic intestinal pseudo-obstruction, total colectomy, distal gastrectomy

## Abstract

**INTRODUCTION:**

Hypoglycemia can lead to significant adverse effects, including cognitive impairment, fatigue, convulsions, and even loss of consciousness in severe cases. Recent reviews also associate hypoglycemia with severe outcomes, such as mortality, dementia, and cardiovascular events. In gastrointestinal surgery, postoperative hypoglycemia related to dumping syndrome is well documented after gastric procedures. However, hypoglycemia in patients who have undergone both gastrectomy and total colectomy is rare, and the underlying mechanisms and effective management strategies remain unclear.

**CASE PRESENTATION:**

The patient, a 46-year-old woman diagnosed with chronic intestinal pseudo-obstruction (CIPO), had a medical history of distal gastrectomy with Billroth-I reconstruction and colostomy. Recently, she underwent total colectomy, after which she began experiencing symptoms of unexplained malaise, depression, and cognitive decline. She received treatment with medication at a neuropsychiatric department to address these symptoms, but they persisted. Strong anxiety and fatigue led her to engage in frequent drug overdose. She then presented to our hospital. Given her history of gastrointestinal surgery, we considered the possibility of nocturnal hypoglycemia and performed continuous glucose monitoring (CGM), which showed marked hypoglycemia. Nutritional therapy to control hypoglycemia effectively improved her condition, resolving drug overdose behavior completely and reducing the dosage of her psychotropic medications by half.

**CONCLUSION:**

This case highlights the diagnostic utility of CGM and the effectiveness of nutritional management in treating hypoglycemia after total colectomy in addition to gastrectomy and provides new insights into the management of similar postoperative cases.

## INTRODUCTION

Dumping syndrome is a well-recognized complication following gastric surgery, characterized by rapid gastric emptying that can lead to hypoglycemic episodes.^1,2)^ The advent of continuous glucose monitoring (CGM) has made it possible to better understand blood glucose fluctuations after gastrectomy, revealing distinct patterns such as transient postprandial hypoglycemia and persistent nocturnal hypoglycemia.^3)^ In diabetes mellitus patients, hypoglycemia has been shown to trigger sympathetic nervous system activation, leading to an increased risk of mortality associated with ischemic heart disease.^4,5)^ Additionally, hypoglycemia is known to cause symptoms such as fatigue and psychiatric disturbances.^6)^

In this case report, we present a patient who initially underwent distal gastrectomy and subsequently total colectomy due to chronic intestinal pseudo-obstruction (CIPO). After these surgeries, the patient reported worsening fatigue and psychiatric symptoms, prompting further evaluation. CGM revealed severe nocturnal hypoglycemia, leading us to hypothesize that the psychiatric symptoms might be related to nocturnal hypoglycemia and planned nutritional therapy aimed at preventing hypoglycemia. Subsequently, the enteral low-carbohydrate formula was administered before sleep and meals to promote a gradual increase in blood glucose levels, resulting in improvements in both psychiatric symptoms and hypoglycemia. This case highlights the diagnostic value of CGM in identifying hypoglycemia patterns in patients with complex surgical histories and provides insights into effective management strategies for postoperative hypoglycemia.

## CASE PRESENTATION

The patient, a 46-year-old woman diagnosed with CIPO,^7–9)^ had previously undergone distal gastrectomy with Billroth-I reconstruction 8 years ago and colostomy 5 years ago. A colostomy was created in the ascending colon due to adhesions in the upper abdomen. She had not experienced dumping symptoms after the gastrectomy. Aside from this gastrointestinal surgery for CIPO, she had no significant medical history, and there was no indication of glucose intolerance. Last year, due to persistent gastrointestinal dysfunction associated with her condition, she underwent total colectomy. The ileostomy was constructed in the terminal ileum and most of the small intestine was preserved. After this surgery, she began to experience an exacerbation of her dumping symptoms as well as unexplained worsening of symptoms, including malaise, depressive episodes, and cognitive decline. At the time, she was taking 750 mg/day of quetiapine and 1.0 mg/day of lorazepam as part of her neuropsychiatric treatment. These symptoms were also accompanied by episodes of drug overdose driven by severe anxiety and fatigue, and her condition remained largely unresolved.

**Fig. 1:**
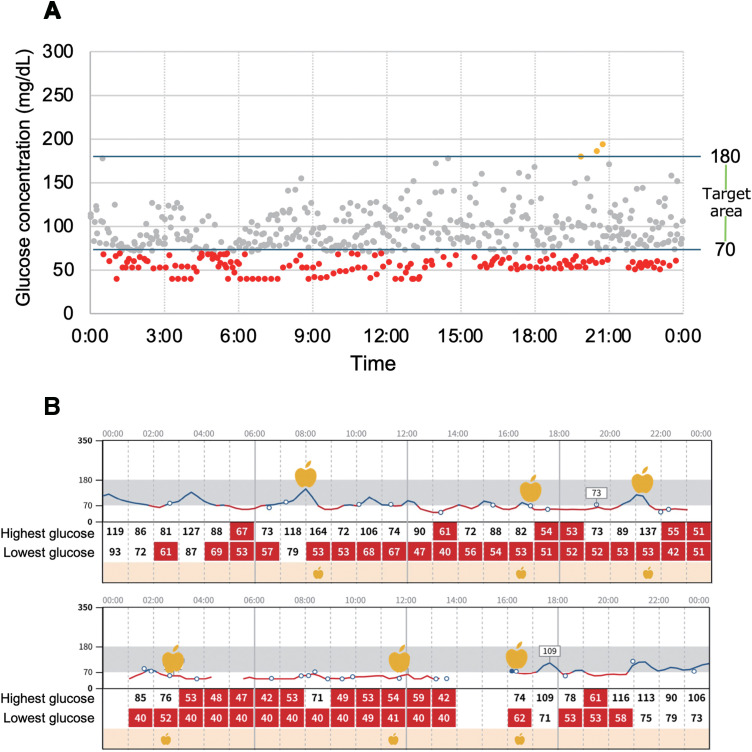
CGM results before the start of nutritional therapy. (**A**) Plots of CGM for 1 week prior to the start of nutritional therapy. Red plots indicate glucose levels less than 70 ml/dL. (**B**) Graphs of diurnal variation in glucose levels on a typical 2 days prior to the start of nutritional therapy. The apple symbols are the time of food intake. It is shown that the patient is in a state of hypoglycemia throughout the day. CGM, continuous glucose monitoring

When she presented to our hospital, we considered her extensive surgical history, including gastrectomy, which is known to predispose patients to prolonged hypoglycemia over time.^2,10)^ It is possible that hypoglycemia had been present since her gastrectomy but became more pronounced following total colectomy, leading to the emergence of symptoms such as fatigue, depressive episodes, and cognitive decline. Given these considerations, we hypothesized that hypoglycemia, potentially exacerbated during nocturnal, could be contributing to her unresolved neuropsychiatric symptoms. To investigate this, we implemented CGM, which indeed revealed significant nocturnal hypoglycemia, but the hypoglycemia persisted not only at night but also during the day. The results supported the lack of diurnal variation in her neuropsychiatric symptoms. For CGM, we used the FreeStyle Libre (Abbott, North Chicago, IL, USA). A plot of glucose values during the first week of CGM is shown in **Fig. 1A**. The red dots represent glucose values below 70 mg/dL, indicating the presence of hypoglycemia. A representative graph of the diurnal variation of glucose values for 2 days is shown in **Fig. 1B**, revealing persistent hypoglycemia both during the night and throughout the day.

**Fig. 2:**
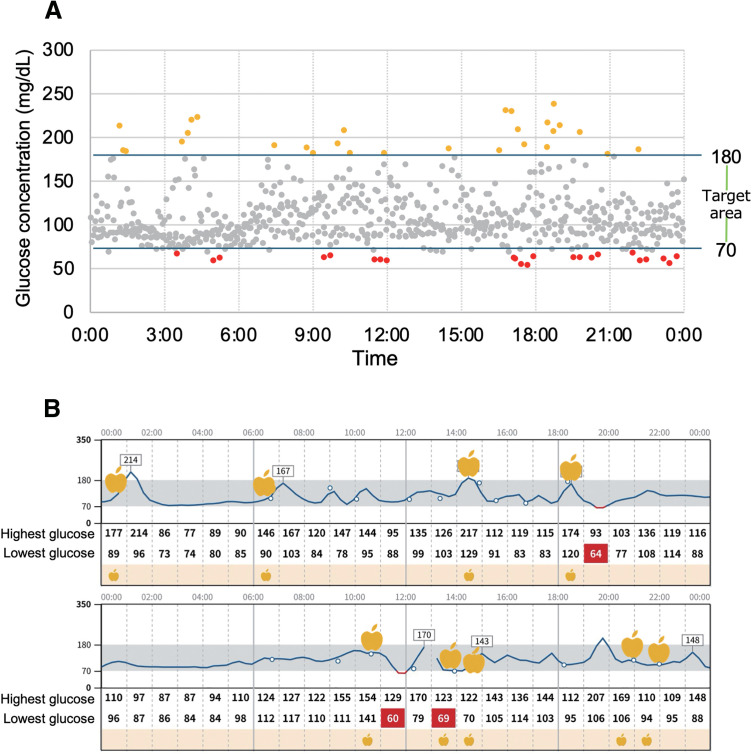
CGM results after initiation of nutritional therapy. (**A**) Plots of CGM for 1 week after the start of nutritional therapy. Red plots indicating hypoglycemia are decreasing. (**B**) Graphs of diurnal variation in glucose levels on a typical 2 days after the start of nutritional therapy. It is shown that the number of meals has increased, and the duration of hypoglycemia has decreased. CGM, continuous glucose monitoring

Based on these findings, we initiated nutritional therapy focused on meal timing and composition to stabilize her glucose levels. Specifically, the patient was instructed to increase the number of meals from three to four or five times per day and to take Glucerna-REX (Abbott), an enteral low-carbohydrate formula selected to help prevent rapid post-meal glucose spikes and the resulting transient hypoglycemia.^11–13)^ To target nocturnal hypoglycemia, she was advised to consume Glucerna-REX before bedtime. Additionally, she was instructed to keep a daily log of meals, intake times, and symptoms. A plot of glucose values for CGM 1 week after the introduction of the nutritional therapy intervention is shown in **Fig. 2A**. As a result, hypoglycemia was markedly reduced overall, and the diurnal variation indicated a shorter duration of prolonged hypoglycemia both at night and during the day (**Fig. 2B**).

**Fig. 3:**
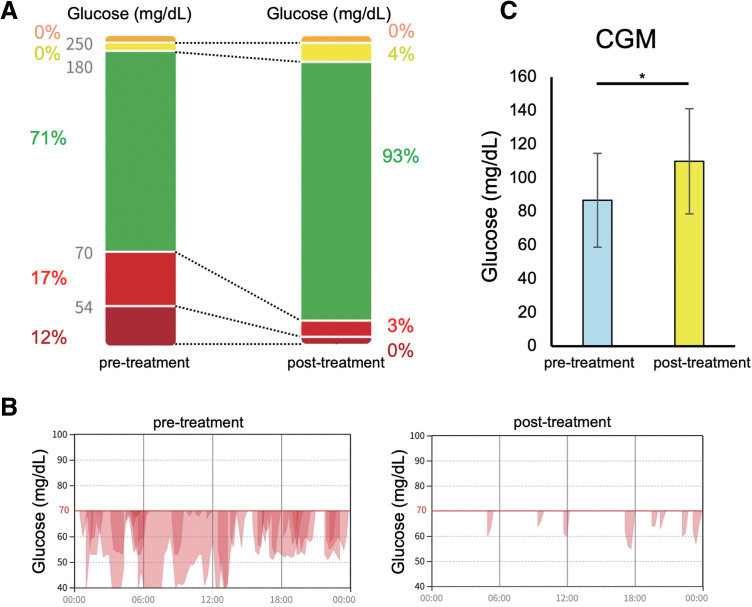
Comparison of glucose levels before and after nutritional therapy. (**A**) Bar graph showing the distribution of glucose levels before and after nutritional therapy. It shows an increase in glucose levels in the target area and a decrease in the percentage of hypoglycemia. (**B**) Graph visualizing the diurnal variation of hypoglycemia (glucose <70 mg/dL). It shows a marked decrease in the duration of hypoglycemia after the start of nutritional therapy. (**C**) Mean and standard deviation of glucose levels before and after the start of nutritional therapy. Significance was calculated by unpaired two-tailed Student’s *t*-test. ^∗^*p* <0.001.

Comparing the 1-week periods before and after the nutritional intervention, the percentage of time with low glucose values (<70 mg/dL) decreased from 29% to 3% (**Fig. 3A**), with marked improvement in nocturnal hypoglycemia (**Fig. 3B**). Additionally, mean (±SD) glucose values significantly increased from 86.7 (±27.9) mg/dL before intervention to 109.9 (±31.3) mg/dL after intervention (**Fig. 3C**). Alongside these improvements in glucose levels, her neuropsychiatric symptoms disappeared, including symptoms of malaise, depressive episodes, and cognitive decline. Her history of drug overdose resolved completely, and the dosage of psychotropic medications was reduced from 750 mg/day of quetiapine and 1.0 mg/day of lorazepam to 400 mg/day of quetiapine and 0.5 mg/day of lorazepam. This led to a notable improvement in her mental and physical health, enabling her to resume Pilates sessions. The final caloric intake was 1000 kcal/day, adjusted by the patient with a nutritional composition of 25% carbohydrate, 25% protein, and 50% fat. Overall, her quality of life markedly improved, and she has continued CGM and nutritional management since then without any recurrence of symptoms.

## DISCUSSION

Hypoglycemia is a well-known complication following gastric surgery, commonly associated with dumping syndrome,^1)^ where rapid gastric emptying leads to postprandial hypoglycemia due to a surge in insulin release.^1,3)^ However, cases involving severe hypoglycemia after both gastrectomy and additional extensive gastrointestinal surgeries, such as total colectomy, are rare and not well understood, with no previous reports to our knowledge.

The patient’s symptoms, including fatigue, depression, and cognitive dysfunction, are typical neuropsychiatric manifestations of hypoglycemia,^14)^ which can greatly impact the quality of life. Hypoglycemia has been associated with severe outcomes, such as cardiovascular events in diabetic patients,^15,16)^ highlighting the importance of prompt recognition and management. These neuropsychiatric effects are often overlooked, especially in patients with complex surgical histories. This case emphasizes the need to consider hypoglycemia in the differential diagnosis of neuropsychiatric symptoms, as timely intervention can lead to rapid symptom relief.

CGM was instrumental in diagnosing hypoglycemia in this patient. CGM provides real-time glucose trends, making it possible to detect hypoglycemic patterns that might be missed with standard intermittent blood glucose measurements.^17)^ Maintaining blood glucose levels within an appropriate range is crucial to prevent adverse events and improve patient outcomes.^18)^ In this case, CGM revealed marked nocturnal and diurnal hypoglycemia that would have been difficult to identify with conventional monitoring alone. This underscores the utility of CGM in diagnosing hypoglycemia in patients with postoperative neuropsychiatric symptoms and highlights its potential for broader use in similar cases.

Nutritional therapy focused on increasing meal frequency and using Glucerna-REX, a high-fat, low-glycemic index supplement, effectively stabilized the patient’s glucose levels. Regular intake of small, low-glycemic meals helps maintain stable glucose levels and prevent hypoglycemic episodes.^18,19)^ In this patient, the intervention not only reduced hypoglycemia but also alleviated neuropsychiatric symptoms, resulting in improved overall quality of life. This case suggests that targeted nutritional therapy may be effective in managing hypoglycemia in postoperative patients who have undergone extensive gastrointestinal surgery, but further research is needed to confirm its efficacy.

In this case, hypoglycemia was likely present but asymptomatic after the gastrectomy, with symptoms emerging after the total colectomy for unclear reasons. Previous reports have described hypoglycemia after gastrectomy as being prolonged at night.^3)^ However, in this case, hypoglycemia persisted during both the daytime and nighttime, with no significant glucose fluctuations after meals. This suggests that reduced glycogen storage capacity may be the underlying cause. Total colectomy results in the loss of gut microbiota-associated metabolic functions, including the production of short-chain fatty acids (SCFAs), which play a crucial role in glucose homeostasis. In particular, propionate serves as a gluconeogenic precursor in the liver, and its depletion following colectomy may lead to reduced gluconeogenesis and impaired glycogen synthesis.^20)^ Furthermore, gut microbiota influences hormone secretion, and a decrease in SCFAs may lead to lower levels of glucagon-like peptide-1 (GLP-1) and peptide YY (PYY).^21)^ GLP-1 regulates insulin secretion, while PYY promotes glycogen synthesis in the liver and muscles. Their decline may contribute to glucose metabolism dysregulation. Additionally, reduced gut fermentation decreases the overall energy supply, potentially leading to diminished glycogen storage in muscles.^22)^ These mechanisms suggest that glucose homeostasis becomes more fragile after total colectomy, increasing the risk of both fasting and postprandial reactive hypoglycemia. Although dietary intervention stabilized blood glucose levels in this case, further studies are needed to assess long-term metabolic adaptations. In addition, other factors such as fluid balance, electrolyte absorption, and body weight changes may contribute to hypoglycemia in patients who undergo total colectomy. The loss of colonic water reabsorption capacity^22)^ may result in chronic dehydration, reducing blood volume and leading to unstable glucose supply to the liver and muscles. Impaired sodium and potassium absorption^20)^ may disrupt insulin secretion and glycogen synthesis. Furthermore, malabsorption and microbiota alterations may affect energy metabolism, leading to weight loss and muscle mass reduction,^21)^ which could further compromise glycogen storage capacity. A combination of reduced glycogen storage capacity and reactive hypoglycemia triggered by meals may have contributed to this condition. Further study is needed to confirm these possibilities.

In summary, this case suggests the importance of considering the possibility of hypoglycemia as a potential cause of neuropsychiatric symptoms in patients with extensive gastrointestinal surgeries. The use of CGM was pivotal in diagnosing the patient’s hypoglycemia and guiding nutritional therapy, underscoring its diagnostic and therapeutic value in postoperative management. Further studies are warranted to clarify the mechanisms of hypoglycemia in patients with combined gastrectomy and colectomy and to develop effective guidelines for prevention and management in these cases.

## CONCLUSION

This case suggests hypoglycemia as a cause of neuropsychiatric symptoms in patients after major gastrointestinal surgeries. CGM enabled timely diagnosis, and nutritional therapy improved glucose stability and symptoms. Proactive glucose monitoring and further research are needed to refine management strategies.

## DECLARATIONS

### Funding

None of the authors received any funding for this study.

### Authors’ contributions

Conceptualization, KO, MN, and NH; data collection, MN; data analysis, KO; validation, SF, TH, HH, and MS; writing—original draft, KO; writing—review & editing, KK, TSat, TSan, YK, TN, KY, and NH; critical revisions, KK, KY, and NH; supervision, MN and NH.

All authors have read and approved the final manuscript. All authors agree to take responsibility for all aspects of the research to ensure the accuracy and integrity of the work.

### Availability of data and materials

All the data in this study are included in this article.

### Ethics approval and consent to participate

Ethical committee approval was not required for this case report. Patient anonymity was preserved, and personal information was protected.

### Consent for publication

Written informed consent was obtained from the patient for the publication of this case report and accompanying images.

### Competing interests

No potential conflicts of interest were disclosed.

## References

[1] van Beek AP Emous M Laville M Dumping syndrome after esophageal, gastric or bariatric surgery: pathophysiology, diagnosis, and management. Obes Rev 2017; 18: 68–85. 27749997 10.1111/obr.12467

[2] Kubota T Shoda K Konishi H Nutrition update in gastric cancer surgery. Ann Gastroenterol Surg 2020; 4: 360–8. 32724879 10.1002/ags3.12351 PMC7382435

[3] Kubota T Shoda K Ushigome E Utility of continuous glucose monitoring following gastrectomy. Gastric Cancer 2020; 23: 699–706. 31916026 10.1007/s10120-019-01036-5

[4] Mattishent K Loke YK. Meta-analysis: association between hypoglycemia and serious adverse events in older patients treated with glucose-lowering agents. Front Endocrinol (Lausanne) 2021; 12: 571568. 33763024 10.3389/fendo.2021.571568 PMC7982741

[5] Christou MA Christou PA Kyriakopoulos C Effects of hypoglycemia on cardiovascular function in patients with diabetes. Int J Mol Sci 2023; 24: 9357. 37298308 10.3390/ijms24119357 PMC10253702

[6] Verhulst CEM Fabricius TW Nefs G Consistent effects of hypoglycemia on cognitive function in people with or without diabetes. Diabetes Care 2022; 45: 2103–10. 35876660 10.2337/dc21-2502 PMC9472511

[7] Stanghellini V Camilleri M Malagelada JR. Chronic idiopathic intestinal pseudo-obstruction: clinical and intestinal manometric findings. Gut 1987; 28: 5–12. 3817584 10.1136/gut.28.1.5 PMC1432727

[8] Stanghellini V Corinaldesi R Barbara L. Pseudo-obstruction syndromes. Baillieres Clin Gastroenterol 1988; 2: 225–54. 3289641 10.1016/0950-3528(88)90029-2 PMC7135556

[9] Mann SD Debinski HS Kamm MA. Clinical characteristics of chronic idiopathic intestinal pseudo-obstruction in adults. Gut 1997; 41: 675–81. 9414977 10.1136/gut.41.5.675 PMC1891582

[10] Kubota T Yubakami M Ushigome E Persistent postgastrectomy hypoglycemia unawareness in patients with gastric cancer unveiled by a prospective study. Ann Surg Open 2022; 3: e135. 37600103 10.1097/AS9.0000000000000135 PMC10431341

[11] Nishiwaki S Fujimoto H Kurobe T Use of a low-carbohydrate enteral nutrition formula with effective inhibition of hypoglycemia and post-infusion hyperglycemia in non-diabetic patients fed via a jejunostomy tube. Intern Med 2020; 59: 1803–9. 32461526 10.2169/internalmedicine.4465-20 PMC7474979

[12] van Meijeren J Timmer I Brandts H Evaluation of carbohydrate restriction as primary treatment for post-gastric bypass hypoglycemia. Surg Obes Relat Dis 2017; 13: 404–10. 27986586 10.1016/j.soard.2016.11.004

[13] Hirose S Iwahashi Y Seo A Concurrent therapy with a low-carbohydrate diet and miglitol remarkably improved the postprandial blood glucose and insulin levels in a patient with reactive hypoglycemia due to late dumping syndrome. Intern Med 2016; 55: 1137–42. 27150868 10.2169/internalmedicine.55.5655

[14] King P Kong MF Parkin H Well-being, cerebral function, and physical fatigue after nocturnal hypoglycemia in IDDM. Diabetes Care 1998; 21: 341–5. 9540013 10.2337/diacare.21.3.341

[15] Desouza C Salazar H Cheong B Association of hypoglycemia and cardiac ischemia: a study based on continuous monitoring. Diabetes Care 2003; 26: 1485–9. 12716809 10.2337/diacare.26.5.1485

[16] Su G Mi S Tao H Association of glycemic variability and the presence and severity of coronary artery disease in patients with type 2 diabetes. Cardiovasc Diabetol 2011; 10: 19. 21349201 10.1186/1475-2840-10-19 PMC3056765

[17] Seidu S Kunutsor SK Ajjan RA Efficacy and safety of continuous glucose monitoring and intermittently scanned continuous glucose monitoring in patients with type 2 diabetes: a systematic review and meta-analysis of interventional evidence. Diabetes Care 2024; 47: 169–79. 38117991 10.2337/dc23-1520

[18] Battelino T Danne T Bergenstal RM Clinical targets for continuous glucose monitoring data interpretation: recommendations from the international consensus on time in range. Diabetes Care 2019; 42: 1593–603. 31177185 10.2337/dci19-0028 PMC6973648

[19] Peng J Lu J Ma X Breakfast replacement with a liquid formula improves glycaemic variability in patients with type 2 diabetes: a randomised clinical trial. Br J Nutr 2019; 121: 560–6. 30526707 10.1017/S0007114518003628

[20] Topping DL Clifton PM. Short-chain fatty acids and human colonic function: roles of resistant starch and nonstarch polysaccharides. Physiol Rev 2001; 81: 1031–64. 11427691 10.1152/physrev.2001.81.3.1031

[21] Clarke G Stilling RM Kennedy PJ Minireview: gut microbiota: the neglected endocrine organ. Mol Endocrinol 2014; 28: 1221–38. 24892638 10.1210/me.2014-1108 PMC5414803

[22] Cummings JH Englyst HN. Gastrointestinal effects of food carbohydrate. Am J Clin Nutr 1995; 61: 938s–45s. 7900692 10.1093/ajcn/61.4.938S

